# Evaluation of toceranib for treatment of apocrine gland anal sac adenocarcinoma in dogs

**DOI:** 10.1111/jvim.15706

**Published:** 2020-01-24

**Authors:** Caitlin M. Heaton, Arthur F. A. Fernandes, Paulo C. Jark, Xuan Pan

**Affiliations:** ^1^ Department of Medical Sciences, School of Veterinary Medicine University of Wisconsin‐Madison Madison Wisconsin; ^2^ Animal Sciences University of Wisconsin‐Madison Wisconsin; ^3^ Universidade Estadual Paulista Julio de Mesquita Filho‐Campus de Jaboticabal Jaboticabal SP Brazil; ^4^ Carbone Cancer Center University of Wisconsin‐Madison Madison Wisconsin

**Keywords:** AGASACA, canine, OST, PFS

## Abstract

**Background:**

There is no widely accepted standard medical treatment for apocrine gland anal sac adenocarcinoma (AGASACA) in dogs. Targeted agents such as toceranib may be effective in treatment of AGASACA, but the number of clinical reports investigating its efficacy is limited.

**Hypothesis/Aim:**

To evaluate the efficacy of toceranib treatment of AGASACA in dogs, and to assess prognostic factors in the study population. Our hypothesis was that toceranib would provide a clinical benefit in the treatment of dogs with AGASACA.

**Animals:**

Thirty‐six client‐owned dogs with either a cytologic or histologic diagnosis of AGASACA that were treated with toceranib alone or in combination with surgery, nonconcurrent chemotherapy or both.

**Methods:**

Retrospective study.

**Result:**

The median progression‐free survival (PFS) and overall survival time (OST) for the study population was 313 days and 827 days, respectively. A clinical benefit from toceranib treatment was observed in 69% of dogs, with 20.7% of dogs experiencing partial response and 48.3% of dogs experiencing stable disease. Dogs that responded to toceranib treatment had significantly prolonged PFS and OST. Hypercalcemia was a negative prognostic factor for clinical outcomes.

**Conclusions:**

Toceranib is effective in the treatment of AGASACA in dogs. Prospective, controlled clinical trials are needed to determine the efficacy of toceranib in comparison to other treatment protocols for dogs with AGASACA.

**Abbreviations**
AE: adverse event
AGASACA: apocrine gland anal sac adenocarcinoma
CB: clinical benefit
CR: complete response
CT: computerized tomography
FDA: U. S. Food and Drug Administration
HR: hazard ratio
KM: Kaplan Meier
MTD: maximum tolerated dose
PDGFR: platelet derived growth factor receptor
PR: partial response
RECIST: Response Evaluation Criteria in Solid Tumors
SD: stable disease
TX: treatment
UPC: urine protein to creatinine ratio
VCOG: Veterinary Comparative Oncology Group
VCOG‐CTCAE: Veterinary Co‐operative Oncology Group's common terminology criteria for adverse events
VEGFR: vascular endothelial growth factor receptor

## 1 INTRODUCTION

Apocrine gland anal sac adenocarcinoma (AGASACA) is a malignant tumor that arises from the epithelium of the anal sac. It represents 2% of all skin tumors and 17% of perianal malignancies in dogs.^1^ Apocrine gland anal sac adenocarcinoma displays aggressive biologic behavior. It is locally invasive and has a metastatic rate of 50% to 90% by the time of diagnosis.^2^, ^3^, ^4^ Metastasis to regional lymph nodes usually occurs before distant metastasis to sites such as the lungs, liver, and spleen.^3^ Polyuria and polydipsia may be present as a consequence of paraneoplastic hypercalcemia, which is reported in approximately 25% to 50% of cases.^3^, ^5^, ^6^ Despite its high propensity to metastasize, this tumor tends to have an overall indolent disease course with median survival times of 1‐2 years with single or multimodal therapeutic modalities, including surgery,^6^, ^7^ chemotherapy,^8^, ^9^ and radiation.^5^ Although numerous chemotherapeutic agents (carboplatin,^8^ actinomycin D,^9^ mioxantrone,^5^ and melphalan^6^) have been investigated, the definitive role of adjuvant chemotherapy in the treatment of AGASACA remains unclear. Alternative treatment options such as targeted therapy also require further assessment to define therapeutic benefit.

Toceranib phosphate (Palladia) is a small molecule tyrosine kinase inhibitor approved by the United States Food and Drug Administration (FDA) for the treatment of high‐grade cutaneous mast cell tumors in dogs.^10^ Toceranib has activity against several members of the split‐kinase family including vascular endothelial growth factor receptor (VEGFR), platelet‐derived growth factor receptor (PDGFR), Kit and Flt‐3.^11^, ^12^ Toceranib's mechanism of action results from competitive blockade of the ATP‐binding site of tyrosine kinase receptors, impairing phosphorylation and downstream signaling.^13^ With both antiangiogenic as well as antitumor effects, toceranib has potential therapeutic activity in a wide range of tumor types in dogs.^14^, ^15^, ^16^, ^17^, ^18^, ^19^, ^20^, ^21^, ^22^, ^23^ A phase I clinical trial and retrospective case series suggest that toceranib exhibits potential clinical antitumor activity against mixed mammary carcinoma, soft tissue sarcoma, multiple myeloma, AGASACA, osteosarcoma, thyroid carcinoma, head and neck carcinoma, and nasal carcinoma.^12^, ^24^

Use of toceranib for treatment of AGASACA has been evaluated in a limited number of studies. In 1 study of 32 dogs with AGASACA, a clinical benefit from toceranib was reported in 87.5% of dogs, with 25% achieving partial response (PR) and 75% experiencing stable disease (SD).^24^ The median duration for PR was 22 weeks and the median duration for SD was 30.5 weeks. A more recent retrospective study evaluated dogs diagnosed with stage 4 AGASACA, and 13 of 15 dogs experienced clinical benefit from toceranib treatment by stabilization of their disease.^25^ Thus, toceranib may provide an attractive adjunctive treatment option for AGASACA in dogs, and further investigation of its effectiveness is needed.

Our primary aim was to retrospectively evaluate the efficacy of adjuvant and primary toceranib treatment in dogs with AGASACA. A secondary aim was to identify prognostic factors in the study population. A clinical benefit was defined as complete response (CR) or PR at any point in time or SD for at least 10 weeks. We hypothesized that toceranib would provide clinical benefit in dogs treated for AGASACA.

## 2 MATERIALS AND METHODS

### 2.1 Case selection and data collection

Dogs with a definitive ante‐mortem diagnosis of AGASACA were identified by reviewing medical records from University of Wisconsin Veterinary Care (UWVC) from 2009 through 2019. Inclusion criteria included either a cytologic or histologic diagnosis of AGASACA for which toceranib was used as either sole, adjuvant, or neoadjuvant treatment. All patients underwent complete staging including history, physical examination, CBC, serum biochemistry profile, urinalysis, 3‐view thoracic radiographs, and abdominal ultrasound or abdominal computerized tomography (CT) scanning. Dogs that had been treated by surgery or nonconcurrent chemotherapy in addition to toceranib also were included in the study. Dogs treated by radiation therapy were excluded.

Restaging by physical examination, thoracic radiographs, and abdominal ultrasound examination was recommended every 2 to 3 months to assess therapeutic response. A CBC was recommended 2 weeks after initiating toceranib to monitor for myelosuppression. Reevaluations including physical examination, CBC, serum biochemistry profile, urinalysis, urine protein‐to‐creatinine ratio (UPC; if indicated), blood pressure measurement, thoracic radiographs and abdominal ultrasound or CT scan of the thorax and abdomen were recommended 1 month after starting toceranib. If toceranib was well tolerated, reevaluations with restaging were recommended every 2 to 3 months while receiving continued treatment. The majority of patients (26/36) were restaged according to these guidelines. A minority of the study population (10/36) was not consistently restaged because of lack of client compliance. All dogs were included on an intent‐to‐treat basis. The standard toceranib (Palladia, Zoetis, Florham Park, New Jersey) protocol consisted of a target dosage of 2.75 mg/kg to be administered PO q48h or on a Monday‐Wednesday‐Friday basis. An altered dosing schedule or a ≥20% dose reduction was employed at the discretion of the primary clinician if adverse events were noted. When performing data analysis, dogs were separated into 3 groups based on toceranib dosage: 2.0 to 2.5 mg/kg, 2.5 to 3.0 mg/kg, and 3.0 to 3.5 mg/kg.

The following information was collected from medical records when available: signalment, body weight, primary tumor size and location, surgical treatment and surgical margins, presence of hypercalcemia, metastatic disease status at the initiation of toceranib treatment, prior or subsequent chemotherapy, toceranib dosage and treatment duration, and adverse events. Surgical margins were defined as complete if neoplastic cells were not present within 5 mm of the cut edge of the excised tumor, based on the original histopathology report. Surgical margins were defined as incomplete if neoplastic cells were present at the cut edge or within 5 mm of the cut edge.

### 2.2 Antitumor response assessment

Tumor response was assessed in 29 patients with macroscopic disease by using the Response Evaluation Criteria in Solid Tumors (RECIST) 1.1.^26^ Tumor assessment was performed using caliper measurement, thoracic radiographs, ultrasonography, or CT as indicated. A CR was defined as complete regression of disease; PR was defined as ≥30% decrease in the sum of the longest diameters of the target lesions, no progression of nontarget lesions, and no new lesions; PD was defined as a >20% increase in the sum of the longest diameters of the target lesions, progression of nontarget lesions or the appearance of a new lesion; SD was defined as absence of CR, PR, or PD for ≥10 weeks. Dogs were defined as experiencing clinical benefit if they had CR, PR at any point in time, or SD for ≥10 weeks.

Progression‐free survival (PFS) was defined as the time of initiation of toceranib treatment to the time of PD or death of any cause. PFS was assessed for the total population (36 dogs), patients with macroscopic disease (29 dogs), and patients with microscopic disease (7 dogs). Overall survival time (OST) was defined as the time of disease diagnosis to the time of death of any cause. Toceranib treatment‐associated OST was defined as the time of initiation of toceranib treatment to the time of death of any cause. Toceranib treatment‐associated OST was assessed for the total population (36 dogs), patients with macroscopic disease (29 dogs), and patients with microscopic disease (7 dogs). Follow‐up information was obtained by medical record review or by contacting the primary care veterinary clinic when necessary.

### 2.3 Assessment of adverse events (AEs)

All patients were evaluated at the UWVC before toceranib treatment. Physical examination, CBC, serum biochemistry profile, urinalysis, and blood pressure measurement were recommended before treatment, 1 month after initiation of toceranib treatment and every 2 to 3 months while receiving toceranib. Urine protein‐to‐creatinine ratio was performed if results of a patient's urinalysis or blood pressure measurement indicated proteinuria or systemic hypertension. All AEs were graded according to the Veterinary Co‐operative Oncology Group's common terminology criteria for AEs (VCOG‐CTCAE v1.1).^27^ For gastrointestinal AEs, supportive care included, but was not limited to, a 7‐day toceranib treatment holiday, antidiarrheal agents (eg, metronidazole, tylosin), antiemetics (eg, maropitant, metoclopramide, ondansetron) and gastric protectants (eg, omeprazole, famotidine). Treatment of hypertension included an angiotensin‐converting enzyme (ACE) inhibitor (eg, enalapril, benazepril), amlodipine, or both.

### 2.4 Statistical analysis

Descriptive statistics were used to summarize the variables collected on the group of patients treated and to define toceranib dosing strata. The Kaplan‐Meier (KM) product of survival probabilities was used to assess PFS and OST curves for the population and for different cohorts of interest. Dogs lost to follow‐up were censored in the survival analysis at the last known follow‐up event. Survival curves were compared using the log rank test. A multivariable Cox proportional hazard model was used to assess the rate of possible death events given the prognostic factors. A *P*‐value for the log rank test of <.05 was considered statistically significant. All analyses were performed in *R*,^28^ and the KM curves and Cox proportional hazard models were performed using the survival package (GraphPad Software, La Jolla, California).^29^

## 3 RESULTS

### 3.1 Study population

Thirty‐six dogs diagnosed with AGASACA and treated with toceranib alone or in combination with surgery, nonconcurrent chemotherapy or both during the study's time period fulfilled the study's inclusion criteria. The population consisted of 11 female dogs and 25 male dogs, and all dogs were either spayed or neutered. Nineteen different breeds were represented (Table 1). Median age at diagnosis was 10 years (range, 6‐15 years) and median body weight was 22 kg (range, 2.4‐58.4 kg). Median primary tumor size was 3 cm (range, 0.2‐10 cm). Most dogs (80.6%) were treated with toceranib in the gross disease setting characterized by the presence of a primary tumor, recurrent tumor, metastatic disease or some combination of these. The remaining dogs (19.4%) were treated with adjuvant toceranib in the postresection microscopic disease setting with no measurable evidence of gross disease. Hypercalcemia was detected in 14 of 36 dogs (38.9%). Metastatic disease was present at initiation of toceranib treatment in most dogs (86.1%), with most metastatic disease located in the regional lymph nodes. Detailed patient clinical characteristics are presented in Table 2.

**Table 1:** Breed summary (n = 36)

Breed	Number of dogs	Percentage (%)
Labrador Retriever	8	22.2
Poodle/X	5	13.9
Golden Retriever	3	8.3
German Shepherd	2	5.6
Border Collie/X	2	5.6
Dachshund	2	5.6
Cocker Spaniel	2	5.6
Bichon	1	2.8
Schnauzer	1	2.8
Boxer	1	2.8
Shetland Sheepdog	1	2.8
Chihuahua	1	2.8
Portuguese Water dog	1	2.8
Sealyham Terrier	1	2.8
Springer Spaniel	1	2.8
Alaskan Malamute	1	2.8
Yorkshire Terrier	1	2.8
Catahoula Leopard Dog	1	2.8
Tibetan Spaniel	1	2.8

**Table 2:** Patient characteristics and adjunctive treatments (n = 36)

Parameter	Number of dogs	Percentage (%)
Sex		
Male castrated	25	69.4
Female spayed	11	30.6
Metastasis		
Absent	5	13.9
Present	31	86.1
Regional lymph node	30	83.3
Lungs	6	16.7
Hypercalcemia		
Yes	14	38.9
No	22	61.1
Additional treatments		
Surgery		
Yes	23	63.9
Complete margins^a^	10	43.5
Incomplete margins^b^	13	56.5
No	13	36.1
Chemotherapy		
Yes	21	58.3
No	15	41.7
Disease setting		
Gross	29	80.6
Micro	7	19.4

a Surgical margins were defined as complete if neoplastic cells were not present within 5 mm of the cut edge of excision, based on the original histopathology report.

b Surgical margins were defined as incomplete if neoplastic cells were present at the cut edge or within 5 mm of the cut edge.

### 3.2 Treatments

The majority of dogs (23/36, 63.9%) underwent surgery for removal of the primary tumor before treatment with toceranib. Regional lymph nodes were removed surgically in 16 dogs and 4 dogs did not have metastatic lymph nodes removed at or after the time of primary tumor resection. Of the 23 dogs treated with surgery, complete surgical excision (ie, complete surgical margins) was accomplished in 10 cases and incomplete surgical margins were noted in 13 cases. Of the 36 dogs in the study population, 10 received chemotherapy before toceranib treatment, 3 received chemotherapy after toceranib treatment because of PD and 8 dogs received chemotherapy both before and after toceranib treatment. The majority of dogs that received chemotherapy received multiple chemotherapy agents. Chemotherapy agents used included carboplatin (n = 16), doxorubicin (n = 7), melphalan (n = 6), mitoxantrone (n = 4), metronomic cyclophosphamide at 14.5 mg/m^2^ once daily (n = 5), lomustine (n = 1), and GS074 (an investigational cytotoxic agent; n = 1). Two dogs received an alternative targeted agent (masitinib) for a period of time after toceranib treatment. Nine dogs received toceranib as sole treatment without surgery or chemotherapy (Table 2).

### 3.3 Toceranib dosing and adverse events

Most dogs (31/36) were treated with toceranib on an every other day basis. Five dogs were started on a Monday‐Wednesday‐Friday dosing schedule. The median initial toceranib dosage was 2.7 mg/kg (range, 2.1‐3.8 mg/kg). The median duration of toceranib treatment was 190 days (range, 26‐743 days).

Adverse events were reported in 52.7% of cases (19/36). The most common AEs were diarrhea and anorexia. Most AEs were either grade 1 or 2 and were adequately managed with supportive medications. No grade 4 or grade 5 AEs were reported in the study population (Table 3). No abnormalities were reported on CBC and serum biochemistry profile except for 1 grade I neutropenia. One grade I proteinuria was noted based on UPC. Dose reductions or an altered dosing schedule were required in 16 cases (44.4%). Toceranib was discontinued because of owner dissatisfaction with AEs in 4 dogs, although all AEs were grade 3 or less. Toceranib treatment was discontinued in 2 dogs because of the cost of the medication.

**Table 3:** Adverse events

Adverse event	Grade 1	Grade 2	Grade 3	Grade 4	Grade 5
Diarrhea	9	6	0	0	0
Anorexia	6	4	0	0	0
Vomiting	1	1	0	0	0
Nausea	1	0	0	0	0
Hypertension	0	1	1	0	0
Proteinuria	1	0	0	0	0
Dehydration	0	1	0	0	0
Hypopigmentation	1	0	0	0	0
Facial alopecia	1	0	0	0	0
Facial erythema	1	0	0	0	0
Lethargy	1	0	0	0	0
Lameness	1	0	0	0	0
Neutropenia	1	0	0	0	0

### 3.4 Treatment outcomes

**Figure 1:**
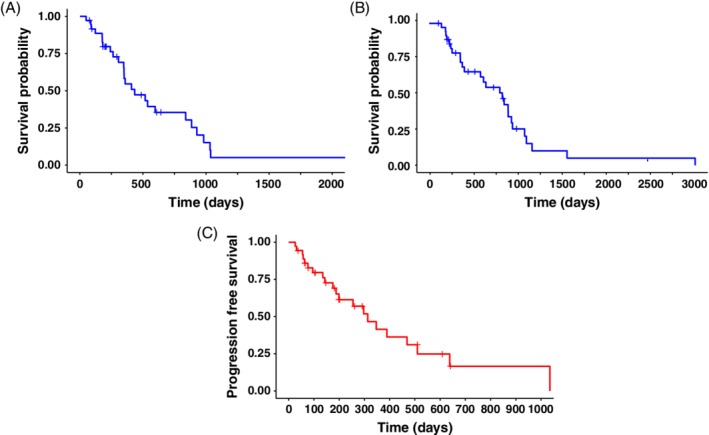
A, Kaplan‐Meier overall survival curve based on the time of starting toceranib treatment (median = 434 days, 49‐2257). B, Kaplan‐Meier overall survival curve based on the time of disease diagnosis. Median = 827 days (138‐3007). C, Kaplan‐Meier progression free survival curve based on the time of starting toceranib treatment. Median = 313 (26‐1035). All censored patients are marked with a cross

**Figure 2:**
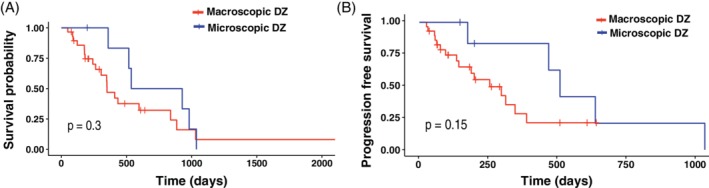
A, Kaplan‐Meier median overall survival curves for patients with microscopic (median = 732 days, 359‐1036) versus macroscopic disease (median = 350 days, 49‐2257). B, Kaplan‐Meier median progression free survival curves for patients with microscopic (median = 510 days, 175‐1035) versus macroscopic disease (median = 255 days, 26‐389). All censored patients are marked with a cross

Toceranib‐associated median OST and the median OST from the time of diagnosis (36 dogs) were 434 days (Figure 1A) and 827 days (Figure 1B), respectively. The PFS (from initiation of toceranib treatment) in the study population was 313 days (Figure 1C). Among 36 patients, 29 received toceranib in the macroscopic disease setting. Based on the KM product of survival probabilities, median PFS and median OST for dogs treated with toceranib in the gross disease setting (29/36) were 255 and 350 days, respectively, compared to 510 and 732 days in the microscopic disease setting (7/36; Figure 2). Among 29 patients with macroscopic disease, clinical benefit was noted in 69% of dogs (20/29); 6 dogs (20.7%) had PR and 14 dogs (48.3%) had SD. Progressive disease was observed in 31% of dogs (9/29).

**Figure 3:**
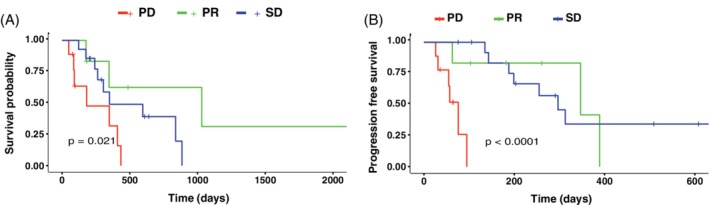
A, Kaplan‐Meier overall survival curve based on clinical response: partial response (PR) (median = 1031 days, 177‐2257), stable disease (SD) (median = 350 days, 122‐1036), and progressive disease (PD) (median = 181 days, 49‐434). B, Kaplan‐Meier progression free survival curve based on PR (median = 347 days, 63‐389), SD (median = 469 days, 135‐1035), and PD (median = 76 days, 26‐95). All censored patients are marked with a cross

Response to treatment was significantly associated with both PFS (*P* < .001) and toceranib‐associated OST (*P* = .02; Figure 3). Median PFS for dogs with PR, SD, and PD was 347 days, 469 days, and 76 days, respectively. Median OST for dogs with PR, SD, and PD was 1031 days, 350 days, and 181 days, respectively. Kaplan‐Meier survival curves for these 3 subsets of the population based on clinical response are shown in Figure 3. Median PFS and OST for dogs treated by surgery were 347 and 517 days, compared to 313 and 350 days for dogs that did not have surgery. Median PFS and OST for dogs with hypercalcemia were 313 and 517 days, respectively, compared to 347 and 409 days for normocalcemic dogs. Median PFS and OST for dogs with metastasis were 313 and 536 days, respectively, compared to 135 and 359 days for dogs without metastatic disease. Median PFS and OST for dogs that received chemotherapy were 313 and 434 days, respectively, compared to 143 and 359 days for dogs that did not receive chemotherapy. The group of dogs that received toceranib within the dosage range of 2.0 to 2.5 mg/kg had median PFS of 297 days and median OST of 981 days. The group of dogs that received toceranib within the dosage range of 2.5 to 3.0 mg/kg had median PFS of 199 days and median OST of 434 days. The group of dogs that received toceranib within the dosage range of 3.0 to 3.5 mg/kg had median PFS of 313 days and median OST of 350 days. Female dogs had median PFS of 347 days and median OST of 517 days. Male dogs had median PFS of 297 days and median OST of 409 days. Differences in median PFS and OST were not statistically significant when comparing patients based on different toceranib dosages, surgical treatment, presence of hypercalcemia, chemotherapy, or sex (Figures S1 and S2). Median PFS and OST for each cohort are listed in Table 4.

**Table 4:** PFS and OST based on KM survival curve and prognostic factors based on Cox proportional hazard model

						Cox proportional analysis			
		KM analysis				PFS		OST	
Variable	Number of dogs	Median PFS min‐max)	Median OST (min‐max)			HR (95% CI)	*P*‐value	HR (95% CI)	*P*‐value
Dosage group									
2.5 mg/kg	11	297 (76‐638)	981 (176‐1031)						
3.0 mg/kg	14	199 (26–1035)	434 (86‐2257)			1.81 (0.42‐7.71)	0.42	0.87 (0.26‐2.90)	0.81
3.5 mg/kg	11	313 (31‐347)	350 (49‐838)			0.55 (0.11‐2.78)	0.47	0.84 (0.20‐3.65)	0.82
Surgery									
Yes	23	347 (26–1035)	517 (49‐2257)			1.35 (0.25‐7.23)	0.73	0.42 (0.07‐2.44)	0.33
No	13	313 (63‐313)	350 (90‐885)						
Hypercalcemia									
Yes	14	313 (26‐638)	517 (49‐1031)			8.33 (1.92‐36.18)	0.004**	4.99 (1.27‐19.65)	0.02*
No	22	347 (55‐1035)	409 (90‐2257)						
TX setting									
Gross	29	255 (26–389)	350 (49‐2257)						
Microscopic	7	510 (175–1035)	732 (359–1036)			0.02 (0.001‐0.3)	0.006**	0.34 (0.07‐1.72)	0.20
Metastasis									
Yes	31	313 (26–1035)	536 (49–2257)			0.05 (0.003‐0.73)	0.03*	0.14 (0.02‐0.89)	0.04*
No	5	135 (55‐NA)	359 (122‐434)						
TX response									
PR	6	347 (63–389)	1031 (177–2257)			0.006 (0.0003‐0.12)	0.001**	0.06 (0.01‐0.44)	0.005**
SD	21	469 (135‐1035)	350 (122–1036)			0.007 (0.0005‐0.10)	0.001**	0.20 (0.04‐1.00)	0.05*
PD	9	76 (26‐95)	181 (49–434)						
Toxicity									
Yes	19	297 (26–1035)	409 (49‐2257)			4.83 (1.08‐21.56)	0.04*	3.60 (1.04‐12.43)	0.04*
No	17	313 (55‐638)	434 (90‐1031)						
Chemotherapy									
Yes	21	313 (26‐1031)	434 (86‐2257)			1.57 (0.30‐8.01)	0.95	1.46 (0.42‐5.03)	0.55
No	15	143 (31‐199)	359 (49–1031)						
Sex									
Female	11	347 (55–638)	517 (90‐981)						
Male	25	297 (26–1035)	409 (49‐2257)			0.70 (0.18‐2.73)	0.53	1.83 (0.49‐6.84)	0.37

* *P* < .05

** *P* < .01

Abbreviation: HR, hazard ratio.

### 3.5 Prognostic factors

Multiple factors were assessed for prognostic significance using a multivariable Cox proportional hazard model. Hypercalcemia and toxicity were found to be negative prognostic factors with regard to PFS and OST. Response to toceranib was positively associated with PFS and OST for dogs with PR and for those with SD. Toceranib treatment in the microscopic disease setting was positively associated with PFS when compared to dogs treated in the macroscopic setting. Toceranib dosage, surgical treatment, sex, and chemotherapy were not prognostic for clinical outcome. Unexpectedly, presence of metastatic disease was positively associated with clinical outcome. This finding likely is a consequence of small sample size (only 5 dogs did not have metastatic disease) and the inherent bias of subset analysis in small retrospective trials (Table 4).

## 4 DISCUSSION

Our primary objective was to assess the efficacy of toceranib in dogs with AGASACA. The demographics of our study population were consistent with previous reports, providing support for an accurate representation of dogs with AGASACA.^1^, ^2^, ^3^, ^4^, ^5^, ^30^ Most dogs treated in the macroscopic disease setting (69%) experienced clinical benefit from treatment (20.7%, PR; 48.3%, SD) and clinical benefit was positively associated with OST and PFS. These response outcomes are consistent with previous reports.^24^, ^25^ Our study further supports use of toceranib in this therapeutic setting.

Toceranib treatment‐associated median OST and PFS were 434 days and 313 days, respectively (Figure 1A and 1C). Half of the study population (50%) received chemotherapy before salvage toceranib treatment, often after having failed several chemotherapy protocols. The general OST for dogs treated with toceranib and chemotherapy (before or after toceranib treatment) is impacted by all treatment modalities and not just toceranib. Therefore, a direct comparison of PFS (after initiation of toceranib) with PFS of other agents used for first‐line adjuvant therapy is not necessarily a valid comparison. Similarly, toceranib treatment associated‐OST (defined from the time of initiation of toceranib treatment) in our study population is not directly comparable to other studies that calculated OST from the initial treatment or disease diagnosis. Thus, we also calculated OST from the time of diagnosis, which was 827 days (Figure 1B). This outcome is more consistent with the previously reported survival times, ranging from 16 to 31 months.^4^, ^5^, ^6^, ^30^

The rate of metastatic disease in our study population (86.1%) was consistent with that of previous reports, but skewed toward the higher end of the range. Again, the majority of dogs in our study were evaluated later in the course of their disease, because toceranib often was used as adjuvant treatment after surgery or salvage treatment after failure of cytotoxic chemotherapy. Therefore, this higher rate of metastasis is likely a result of selection bias. It is also possible that the toceranib‐associated survival time (434 days) in our study was falsely decreased because of selection bias and the advanced extent of disease in most patients.

A secondary aim of our study was to identify prognostic factors in the study population. Multivariate subgroup analysis, however, can be subject to bias and statistical concerns when used in small cohorts and in retrospective analysis.^31^ Our multivariate Cox hazard analysis suggests that clinical benefit from toceranib treatment was a positive prognostic factor for clinical outcome, but toceranib dosage was not found to be prognostic (Table 4). The original label dosage for toceranib was 3.25 mg/kg q48h, which was first established in the phase I clinical trial to determine the maximum tolerated dose (MTD).^12^ Several more recent reports show that dosages of toceranib ranging from 2.4 to 2.9 mg/kg q48h provide exposure considered sufficient for target inhibition.^12^, ^24^, ^32^ The lower dosage of toceranib, although still effective, also may have fewer AEs, which is advantageous. In our study, no significant difference was found in PFS or OST among toceranib dosage groups. Therefore, our results further support a lower than MTD toceranib dosing protocol for treating dogs with substantial AE profiles.

Based on the multivariable Cox hazard model, toceranib treatment in the microscopic disease setting was associated with prolonged PFS but not with OST (Table 4). Prolonged survival in dogs that had surgical treatment of AGASACA has been well documented in the veterinary literature.^6^, ^7^, ^33^, ^34^, ^35^ Furthermore, positive outcomes have been reported in dogs treated surgically, even in cases of metastatic disease.^30^, ^35^ In our study, only 7 dogs were treated in the microscopic disease setting. The small sample size and difficulty of true assessment of response in the microscopic disease setting restricted interpretation of our data, and therefore these findings should not be used to contradict the current gold standard treatment approach of aggressive surgical treatment, even in face of regional or distant metastasis. Surgical extirpation of metastatic lymph nodes should still be considered in patients that develop progressive metastatic disease later in their clinical course.

Hypercalcemia also was found to be a negative prognostic factor with regard to clinical outcome (Table 4). The prevalence of hypercalcemia (38.9%) in our study was similar to findings in previous reports, with hypercalcemia noted in 25%‐50% of dogs with AGASACA.^3^, ^5^, ^6^ The prognostic relevance of hypercalcemia in AGASACA is discordantly reported in the veterinary literature.^3^, ^5^, ^30^ Multiple studies have found no statistical significance associated with hypercalcemia.^3^, ^5^, ^30^ However, other studies have found hypercalcemia to be a negative prognostic factor associated with shorter survival.^4^ It is possible that interactions exist between hypercalcemia and other prognostic variables, which may explain the presence of prognostic significance in the multivariate Cox analysis but not in the KM analysis.

Our study was a retrospective analysis of observational clinical data. Therefore, imbalance across the variables studied and the presence of missing data are to be expected because patient restaging was inconsistent and several patients were lost to follow‐up. Thirteen dogs were censored in the PFS analysis (including both macroscopic and microscopic disease settings) and 7 dogs were censored in the OST analysis (including both macroscopic and microscopic disease settings) because of lack of follow‐up data. Also, few dogs (n = 7) were included in PFS and OST analysis for dogs treated in the microscopic disease setting. Furthermore, dogs in our study often received multiple treatment modalities including surgery and chemotherapy, in addition to toceranib. Among patients treated by chemotherapy, a variety of drugs and protocols were used. Thus, it is difficult to compare differences and interactions among these various treatment protocols. A larger database may be required for more thorough analysis, which would demand a collaborative effort with other veterinary hospitals.

## 5 CONCLUSIONS

A clinical benefit from toceranib treatment was observed in the majority of dogs in our study (69%). Of dogs that experienced clinical benefit, most experienced stabilization rather than regression of their disease. Toceranib treatment‐associated OST and PFS were 434 and 313 days, respectively. Response to toceranib treatment was associated with both improved OST and PFS. These results support the clinical efficacy of toceranib in the treatment of dogs with AGASACA. Prospective, controlled clinical trials are needed to further evaluate the efficacy of toceranib in comparison to other treatments for dogs with AGASACA.

## CONFLICT OF INTEREST DECLARATION

Authors declare no conflict of interest.

## OFF‐LABEL ANTIMICROBIAL DECLARATION

Authors declare no off‐label use of antimicrobials.

## INSTITUTIONAL ANIMAL CARE AND USE COMMITTEE (IACUC) OR OTHER APPROVAL DECLARATION

Authors declare no IACUC or other approval was needed.

## HUMAN ETHICS APPROVAL DECLARATION

Authors declare human ethics approval was not needed for this study.

## Supporting information


**Supplementary Figure 1** Kaplan‐Meier overall survival curve of patients based on toceranib dosage (**A**); toceranib treatment associated toxicity (**B**); surgical treatment (**C**); presence of hypercalcemia (**D**); presence of metastasis (**E**); gender (**F**); chemotherapeutic treatment. All censored patients are marked with a cross.

**Supplementary Figure 2** Kaplan‐Meier progression free survival curve of patients based on toceranib dosage (**A**); toceranib treatment associated toxicity (**B**); surgical treatment (**C**); presence of hypercalcemia (**D**); presence of metastasis (**E**); gender (**F**); chemotherapeutic treatment (**G**). All censored patients are marked with a cross.
